# Unmet need for modern contraception by HIV status: findings from community—based studies implemented before and after earlier ART initiation program in rural Tanzania

**DOI:** 10.1186/s12978-023-01695-9

**Published:** 2023-10-16

**Authors:** Denna Mkwashapi, Jenny Renju, Michael Mahande, Alison Wringe, John Changalucha, Mark Urassa, Jim Todd

**Affiliations:** 1 Department of Sexual and Reproductive Health, National Institute for Medical Research, Mwanza, United Republic of Tanzania; 2 Department of Population Health, London School of Hygiene and Tropical Medicine, London, UK; 3 Department of Epidemiology and Biostatistics, Kilimanjaro Christian Medical University College, Moshi, United Republic of Tanzania

## Abstract

**Background:**

Tanzania Health policy insists on the need to provide all women access to contraception despite HIV status. We used data from two HIV epidemiologic serological surveys carried out at different periods of ART provision in rural Tanzania to assess the level of unmet need for modern contraception by HIV status and associated factors.

**Methods:**

We performed secondary data analysis of two surveys conducted at the Magu Health and Demographic Surveillance System site, in Mwanza, Tanzania. Information on unmet need for modern contraception was derived from fertility desire and contraception use. Unmet need, HIV status, and socioeconomic and demographic variables were analysed. The percentage of women with unmet needs for modern contraception by HIV status is presented for the 2012 and 2017 surveys. Bivariate and multivariate analyses using logistic regression were used to investigate associated factors showing adjusted odds ratios (aORs) and 95% confidence intervals (95% CIs).

**Results:**

Data from 3352 and 3196 women aged 15–49 years collected in the 2012 and 2017 surveys, respectively, were analysed. The percentages of women with unmet needs for modern contraception in the 2012 and 2017 surveys were 30.9% (95% CI 29.4–32.6) and 31.6% (95% CI 30.0–33.3), respectively. The unmet need for modern contraception was 26% lower in HIV-uninfected women in 2012 (aOR = 0.74; 95% CI 0.569–0.973); p = 0.031). Risk factors for unmet need for modern contraception in 2012 were HIV uninfected (adjusted OR = 0.74; 95% CI 0.569–0.973); p = 0.031), married marital status (adjusted OR = 0.768; 95% CI 0.743–0.794); p < 0.0001), higher education (adjusted OR = 0.768; 95% CI 0.743–0.794); p < 0.0001), and taking alcohol (adjusted OR = 0.768; 95% CI 0.743–0.794); p < 0.0001). Only two factors were associated with unmet need for modern contraception in 2017: married marital status (adjusted OR = 0.46; 95% CI 0.305–0.722); p = 0.001) and women who earned for their families (aOR = 0.66; 95% CI 0.494–0.887); p = 0.006).

**Discussion:**

Nearly one-third of women had an unmet need for modern contraception, which was lower in HIV-uninfected women than in WLHIV-infected women. The study has identified women whose demand for contraception has not been met: WLHIV, post marital women, women with low education and women who were reported to earn money for their families. Family planning interventions should be tailored to these groups of women.

## Introduction

The decision or desire to limit pregnancy and contraception use underpins the concept of unmet need for contraception. Unmet need for contraception is the percentage of reproductive women who are married or sexually active, not pregnant, reported not to desire a child but were not consistently using contraception [1]. It is the percentage of women who would need contraception but they do not. The unmet need for contraception shows the gap between fertility desire and contraception use. It is currently a reliable indicator for universal access to contraception. Higher levels of unmet need for contraption contribute to increased incidences of unintended pregnancies and childhood pregnancies that normally result in abortions and increased maternal death [2].

In Sub-Saharan Africa, 25% of women aged 15–49 years were reported to have an unmet need for female contraception in 2018 [3]. Tanzania DHS has shown that 22% of married women aged 15–49 years have an unmet need for family planning, with regional variations from a low of 10 percent in the Lindi region to a high of 37 percent in the Northern Pemba region [4]. Factors that are associated with changes in the levels of unmet need for family planning were age, number of living children, family size, education, social economic status of the family, opinion of the partner or spouse, availability of family planning service and ethnicity or social structure [5–10].

HIV serostatus plays a significant role in deciding female desire for fertility and contraception use and hence the unmet need for contraception. Women living with HIV (WLHIV) reported greater use of FP services than HIV-uninfected women [7, 11]. Studies have also shown that WLHIV has a greater desire to stop childbearing than HIV-uninfected women [2, 8, 12–14]. However, research using DHS data in nine African countries showed that there is no consistent pattern of fertility desires and contraceptive use among WLHIV when compared to HIV-uninfected women [15].

Recent studies have shown that the levels of unmet need for contraception among WLHIV vary with increased antiretroviral treatment (ART) use. ART use has been a factor not only in reducing the risk for vertical transmission but also in motivating WLHIV to influence their pregnancy decisions despite their HIV sero-positive diagnosis [14].

In 2013, the Prevention of Mother to Child HIV Transmission (PMTCT) program ensured that lifelong free ART was given to all pregnant women diagnosed with HIV regardless of their disease stage and viral or CD4 cell counts [16]. Similarly, the universal HIV test and treat (UTT) policy provided ART to all HIV-infected individuals regardless of their immune status later in 2016 [17, 18]. The Tanzania National Family Planning Research Agenda 2013–2018 insisted on the need to provide all reproductive women access to contraception [19].

It is unclear whether the increased use of ART has impacted the levels of unmet need for contraception among WLHIV. In this paper, we present results from secondary data analysis of two community-based studies carried out in 2012 and 2017. We report the level of unmet need for family planning by HIV status before and after an earlier ART initiation program in rural Tanzania.

## Method

### Study setting

The study was performed at the Magu Health and Demographic Surveillance System site, commonly known as the Magu HDSS site. The site is located in the Magu District, Mwanza region, Tanzania. The research area covers nine villages within three administrative wards, the Kisesa, Bujora and Bukandwe wards, and lies 20 km east of Mwanza city, the regional capital.

By 2020, the Magu HDSS site had a population of approximately 45,000 people. The majority of the population (70%) resided in rural areas. The main social economic activities are small-scale farming, livestock keeping and small-scale petty trading. Government and private sector employees are forming part of the population.

The study population within the area has received health services from five state-owned and one privately owned primary health facility. Free HIV tests, care and treatment services, including PMTCT, reproductive health and family planning services, have been provided at the wards’ referral health centre and four village dispensaries.

### Study design

This was a series of analytical cross-sectional studies that were carried out in 2012 and 2017 as part of the parent study described in the next section.

### Sample size justification

A cross-sectional study with 3000 women of reproductive age per survey will have 90% power to detect a 10% difference in the unmet need for contraception (HIV positive vs. HIV negative) at the 0.05 confidence level [20].

### Data source

HIV serological surveillance system surveys (Sero Survey), which were carried out in 2012 and 2017, were the source of data for this analysis. Data for research purposes were accessed for the first time in December 2019. The Sero survey is another research activity that has been conducted in the Magu HDSS research area since 1994. It is referred to as a sero survey. The Sero survey is a population-based, cross-sectional study that has been implemented in the HDSS population since 1994. It has been repeated every 5 years depending on funds availability. During the Sero survey, village clinics are constructed temporarily in household neighborhoods of the targeted HDSS population. Eligible study participants from the HDSS population are invited to take in the sero surveys. Eligibility criteria for sero survey participation are being residents of HDSS sites and aged 15 years and above. Magu HDSS defines residency as living for three months or more in the study.

From 1994 to 2018, eight rounds of sero surveys were conducted, of which the seventh (2012) and eighth (2017) rounds were used to provide data for this analysis. The 2012 and 2017 surveys were conducted in the HDSS population from December 2012 to July 2013 and from September 2016 to February 2017, respectively. For analysis purposes, we analysed women who were residents of the HDSS site and aged 15–49 years at the time of data collection.

After consenting to participate in the research, the participants underwent a face-to-face interview and provided blood for anonymous HIV testing with or without learning the results. Blood samples were taken in the field and transported to the National Institute for Medical Research (NIMR) laboratory in Mwanza for HIV testing and storage. HIV testing was carried out through the recommended Tanzanian protocol for HIV testing [21]. Details on HIV serological surveillance systems and HIV testing procedures have been described elsewhere [22, 23].

### Variables

In this analysis, data were extracted from two sero survey rounds (2012 and 2017 surveys). Current use of contraception and women’s fertility desire were used to compute the measure for the unmet need for modern contraception. Unmet need for modern contraception was defined as a proportion in which the denominator is all women who are sexually active in the past month, not pregnant, and reported not desire a child in the next two years. Therefore, the unmet need for modern contraception was the proportion of those who were not consistently using modern contraception. The definition of fertility desire used in this paper is described elsewhere [24].

Demographic details of the participants were collected through the standard sero survey questionnaire. The questionnaire collected information on age, HIV status, marital status, education level, residence, occupation, religion, ethnicity, alcohol drinking and cigarette smoking. Other exposure variables were the number of previous pregnancies and ever born living biological children for the women under study. The main outcome variable was unmet need for modern contraception. The variable was derived from information on women’s desire to stop childbearing and their current use of modern contraception. The exposure of interest variable was HIV sero-status. Data entry and management were performed using the Census and Survey Processing System [CSPro software] version 6.3.

### Statistical methods

In the preliminary analysis, we reported the prevalence of unmet needs for modern contraception in the 2012 and 2017 surveys with their 95% confidence intervals. We similarly reported the prevalence of unmet need for contraception by HIV status. We used logistic regression to determine the association between unmet need for contraception and HIV infection. We calculated their associated crude and adjusted odds ratios (ORs) and their 95% confidence intervals. The analysis was performed separately for the 2012 and 2017 surveys. Analysis was performed by using Stata, version 13.0 (Stata Corp, College Station, TX) statistical package.

## Results

A total of three thousand, three hundred fifty-two (3352) and three thousand, one hundred ninety-six (3196) women aged 15–49 years were included in the 2012 and 2017 surveys, respectively. Out of 3352 women in the 2012 survey, 414 (12.4%) were living with HIV**,** 2140 (64.4%) were residing in rural areas and 1895 (56.5%) were reported to complete primary level of education with only 449 (13.4%) reported to attend secondary school education.

Out of 3196 women in the 2017 survey, 237 (7.4%) were living with HIV**,** 1841 (57.6%) were residing in rural areas and 1684 (52.7%) completed a primary level of education, with only 642 (20.01%) attending secondary school education. Details on the characteristics of the women in each survey are shown in Table 1.

**Table 1:** Unmet need for Contraception in women of reproductive age by the year of the survey

Variable	Category	2012 Survey				2017 Survey			
		Number	% Unmet need for Contraception (95%CI)		P value	Number	% Unmet need for Contraception (95%CI)		P value
Women (15–49)		3352	31	(29.43–32.56)		3196	31.63	(30.02–33.25)	
HIV status									
HIV-positive		414	41.3	(27.89–31.19)		237	45.15	(38.77–51.53)	
HIV-negative		2938	29.5	(27.89–31.19)	< 0.001	2959	30.55	(28.89–32.21)	< 0.001
Age group in years									
15–19		768	7.1	(5.33–8.99)		731	24.62	(21.49–27.75)	
20–24		531	8.85	(6.42–11.27)		564	6.56	(4.51–8.61)	
25–29		517	19.15	(15.75–22.55)		490	16.12	(12.86–19.39)	
30–34		502	30.08	(26.05–34.11)		444	22.75	(18.83–26.66)	
35–39		436	47.71	(43.00–52.41)		400	43.25	(38.37–48.13)	
40–44		320	72.19	(67.25–77.12)		326	69.33	(64.29–74.36)	
45–49		278	89.21	(85.54–92.88)	< 0.001	241	89.21	(85.27–93.16)	< 0.001
Place of residence									
Rural		2142	29.88	(27.94–31.82)		1841	31.07	(28.95–33.19)	
Peri Urban		1210	32.98	(30.32–35.63)	0.063	1355	32.39	(29.90–34.89)	0.425
Marital status									
Never married		859	11.53	(9.39–13.66)		878	25.97	(23.06–28.87)	
Currently married		2116	34.17	(32.15–36.19)		1940	30.26	(28.21–32.30)	
Post marriage		377	57.56	(52.55–62.57)	< 0.001	378	51.85	(46.79–56.91)	< 0.001
Education level									
None		820	44.02	(40.06–47.43)		728	39.56	(36.00–43.12)	
Primary (1–4)		188	37.77	(30.77–44.76)		142	33.1	(25.26–40.93)	
Primary (5–7)		1895	29.76	(27.70–31.82)		1684	31.71	(29.49–33.94)	
Secondary and tertiary		449	9.58	(6.84–12.31)	< 0.001	642	22.12	(18.90–25.34)	< 0.001
Religion									
Christian		3096	30.3	(28.68–31.92)		2914	30.82	(29.14–32.49)	
Non-Christian		256	39.45	(33.43–45.48)	0.002	282	40.07	(34.32–45.83)	0.001
Ethnicity									
Sukuma		3137	31.27	(29.65–32.90)		2933	31.98	(30.29–33.67)	
Other		215	26.98	(21.00–32.96)	0.188	263	27.76	(22.31–33.20)	0.158
Earning money									
No		1045	19.43	(17.02–21.83)		1005	30.05	(27.21–32.89)	
Yes		2307	36.24	(34.27–38.20)	< 0.001	2191	32.36	(30.40–34.32)	0.192
Ever been pregnancy									
No		739	7.85	(5.90–9.79)		686	27.55	(24.20–30.90)	
Yes		2613	37.54	(35.69–39.40)	< 0.001	2510	32.75	(30.91–34.59)	0.005
Current alcohol taking									
No		3249	31.02	(29.43–32.62)		3060	31.14	(29.50–32.79)	
Yes		103	30.09	(21.08–39.11)	0.841	136	42.65	(34.23–51.07)	0.005
Current cigarrete smoking									
No		3335	30.88	(29.32–32.45)		3173	31.71	(30.09–33.32)	
Yes		17	52.94	(26.49–79.39)	0.05	23	21.74	(3.50–39.98)	0.306
Number of children									

### The level of unmet need for modern contraception

**Fig. 1:**
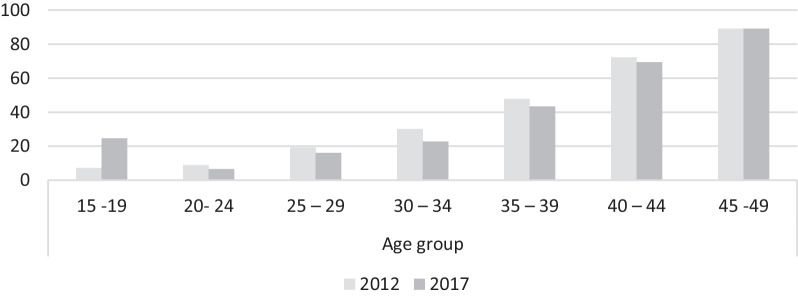
Unmet neeed for modern contraception by age group and year of the survey

Unmet needs for modern contraception in women aged 15–49 years were 30.9% (95% CI 29.4–32.6) and 31.6% (95% CI 30.0–33.3) in the 2012 and 2017 surveys, respectively. In the 2012 survey, the unmet need for modern contraception in women by age group was as low as 7.1% (95% CI 5.3–8.9) in those aged 15–19 years and as high as 89.2% (95% CI 85.0–92.9) in those aged 45–49 years. Similarly, in the 2017 survey, the estimate by age group was as low as 24.6% (95% CI 21.5–27.8) in those aged 15–19 years to as high as 89.2% (95% CI 85.3–93.1) in those aged 45- 49 years. Overall, the unmet need for modern contraceptives tended to increase with increasing age in both surveys (Fig. 1). In the 2017 survey, the unmet need for modern contraception was 24% (95% CI 21.49–27.75) among women of adolescent age [2, 8, 14–16]. This level is higher than the subsequent middle-aged women.

Women with the highest education achievement represented the group of women with the lowest unmet need for modern contraceptives in both surveys. The levels of unmet need for contraceptives among highly educated women rose from 9.6%% (95% CI 6.8–12.3) in 2012 to 22.1% (95% CI 18.9–25.3) in 2017.

Women who were described as “braid winners” for their families were found to have an increased level of unmet need for contraceptives compared to women who were not earning for their family in both surveys. In 2012, the unmet need for modern contraception was 37.5% (35.7–39.4), P < 0.0001, while in 2017, it was 32.7% (30.9–39.5), P < 0.0001. Details on the estimated levels of unmet need for modern contraception are shown in Table 1.

### Unmet need for modern contraception: crude and age-adjusted analysis.

**Fig. 2:**
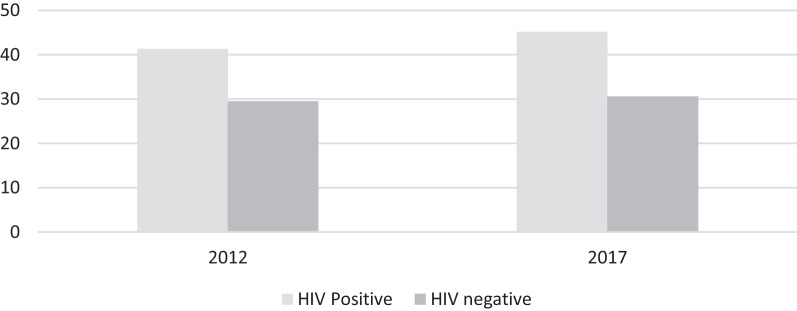
Unmet need for modern contraception by HIV status and year of the survey

The unmet need for contraceptives in WLHIV aged 15–49 years was 41.3% (95% CI 34.53–45.29) in the 2012 survey, while in HIV-uninfected women, the unmet need for contraceptives was 29.5% (95% CI 27.89–31.19). Similarly, in the 2017 survey, the unmet need for contraceptives in WLHIV was 45.5% (95% CI 38.77–51.53), while in HIV-uninfected women, the unmet need for contraceptives was 30.5% (95% CI 28.89–32.21). In comparison, the level of unmet needs for contraception was higher in WLHIV than in their counterparts’ women in both surveys (Fig. 2).

On crude analysis, unmet need for modern contraception in HIV-uninfected women was reduced by 40% compared to unmet need for modern contraception in WLHIV (OR = O.6, 95% CI 0.482–0.736, p < 0.001). A similar analysis in 2017 showed that the unmet need for contraception in HIV-uninfected women was nearly halved compared to the unmet need for contraception in WLHIV (OR = O.5, 95% CI 0.409–0.698, p < 0.001). (Table 2).

**Table 2:** Factors associated with unmet need for contraception in women of reproductive age by year of survey: Crude and age-adjusted analysis

Variable/category	2012 Survey											
	Crude analysis			Age adjusted analysis			Crude analysis			Age adjusted analysis		
	OR	95% Confidence Interval (95%CI)	P value	OR	95% Confidence Interval (95%CI)	P value	OR	95% Confidence Interval (95%CI)	P value	OR	95% Confidence Interval (95%CI)	P value
HIV status												
HIV-Positive	Ref											
HIV-negative	0.6	0.482–0.736	< 0.001	0.86	0.669–1.106	0.241	0.53	0.409–0.698	< 0.001	0.95		0.743
Age group in years												
15–19	Ref						ref					
20–24	1.26	0.839–1.889	0.267				0.21	0.148–0.312	< 0.001			
25–29	3.07	2.161–4.361	< 0.001				0.59	0439–0.789	< 0.001			
30–34	5.58	3.993–7.789	< 0.001				0.9	0.682–1.190	0.465			
35–39	11.8	8.481–16.491	< 0.001				2.33	1.799–3.025	< 0.001			
40–44	33.7	23.300–48.588	< 0.001				6.92	5.180–9.240	< 0.001			
45–49	107	67.131–171.076	< 0.001				25.31	16.296–39.318	< 0.001			
Place of Residence												
Rural												
Peri Urban	1.15	0.992–1.343	0.063	0.92	0.763–1.107	0.375	1.06	0.915–1.236	0.425	0.97	0.816–1.162	0.771
Marital status												
Never married												
Currently married	3.98	3.172–3.172	< 0.001	0.8	0.571–1.135	0.216	1.24	1.034–1.480	0.02	0.45	0.324–0.613	< 0.001
Post marriage	10.4	7.771–13.950	< 0.001	1.21	0.802–1.841	0.357	3.07	2..386–3.949	< 0.001	0.63	0.425–0.940	0.024
Education level												
None												
Primary (1–4)	0.77	0.557–1.068	0.118	0.82	0.556–1.216	0.327	0.76	0.517–1.105	0.149	0.9	0576–1.412	0.652
Primary (5–7)	0.54	0.455–0.638	< 0.001	0.74	0.602–0.910	0.004	0.71	0.592–0.849	< 0.001	0.9	0.727–1.123	0.363
Secondary and tertiary	0.13	0.096–0.190	< 0.001	0.51	0.348–0.760	0.001	0.43	0.342–0.551	< 0.001	1.02	0.759–1.388	0.872
Religion												
Christian												
Non-Christian	1.5	1.153–1.948	0.002	1.3	0.945–1.800	0.106	1.5	1.168–1.929	0.002	1.4	1.048–1.881	0.023
Ethnicity												
Sukuma												
Other	0.81	0.595–1.107	0.188	0.8	0.554–1.149	0.226	0.82	0.617–1.082	0.159	0.9	0.618–1.183	0.345
Earning Money												
No												
Yes	2.36	1.978–2.809	< 0.001	0.8	0.632–1.015	0.067	1.11	0.947–1.309	0.192	0.52	0.414–0.657	< 0.001
Ever been pregnancy												
No												
Yes	7.06	5.337–9.334	< 0.001	1.94	1.261–2.989	0.003	1.28	1.062–1.544	0.01	0.38	0.260–0.556	< 0.001
Current alcohol taking												
No												
Yes	0.96	0.624–1.468	0.841	0.3	0.180–0.507	1.64	1.160–2.329	0.005	0.96	0.622–1.484	0.856	Yes
Current cigarrete smoking												
No												
Yes	2.51	0.969–6.543	0.058	1.02	0.338–3.138	0.959	0.6	0.222–1.616	0.311	0.51	0.165–1.609	0.254
Number of children	1.39	1.331–1.446	< 0.001	1.19	1.131–1.26	1.46	1.403–1.535	< 0.001	1.26	1.194–1.336	< 0.001	

### Factors associated with unmet need for modern contraception: multivariable analysis

We finally conducted the multivariable analysis using logistic regression to explore association between the unmet need for modern contraception and associated risk factors. In this model we adjusted for age, education level, place of residence, reports on personal earning for the family, alcohol drinking and number of living children. Unmet need for modern contraception was associated with HIV infection in the 2012 survey (adjusted OR = 0.74; 95% CI 0.569–0.973); p = 0.031). A similar analysis for the 2017 survey, the unmet need for modern contraception was not found to be associated with HIV infection (adjusted OR = 0.78; 95% CI 0.559–1.126; p = 0.196).

In the 2012 survey, we found the following factors to be associated with increased level of unmet need for modern contraception: increasing age (adjusted OR = 2.14; 95% CI 1.899–2.193); p < 0.0001) and increasing number of living children (adjusted OR = 1.23; 95% CI 1.163–1.300); p < 0.0001). However, the following factors were found to decrease the level of unmet need for modern contraception: HIV infection (adjusted OR = 0.74; 95% CI 0.569–0.973); p = 0.031), currently married status against single women (adjusted OR = 0.768; 95% CI0.743–0.794); p < 0.0001), higher education against those who had not been to school (adjusted OR = 0.768; 95% CI0.743–0.794); p < 0.0001), and women who were taking alcohol (adjusted OR = 0.768; 95% CI0.743–0.794); p < 0.0001).

Similar analysis on the 2017 survey data, only two factors were found to increase the level of unmet need for modern contraception: increasing age (adjusted OR = 2.09; 95% CI 1.897–2.193); p < 0.0001) and increasing number of living children (adjusted OR = 1.34; 95% CI 1.260–1.423); p < 0.0001). However, the following factors were found to decrease the level of unmet need for modern contraception: currently married status against single women (adjusted OR = 0.46; 95% CI 0.305–0.722); p = 0.001) and women who were reported to earn money for their families (adjusted OR = 0.66; 95% CI 0.494–0.887); p = 0.006).The final Multivariable model for both surveys, adjustment was for age, education level, place of residence, reports on personal earning for the family, alcohol drinking and number of living children (Table 3).

**Table 3:** Factors associated with unmet need for contraception in women of reproductive age by year of the survey: Adjusted analysis

	2012 Survey			2017 Survey		
	Adjusted OR	95% Confidence intervals (95%CI)	P value	Adjusted OR	95% Confidence intervals (95%CI)	P value
HIV status						
HIV-Positive	ref			Ref		
HIV-Negative	0.74	0.567–0.973	0.031	0.78	0.559–1.126	0.196
Age	2.14	1.992–2.303	< 0.001	2.09	1.897–2.193	< 0.001
Residence						
Rural	ref			Ref		
Urban	1.11	0.899–1.369	0.333	1.19	0.953–1.496	0.122
Marital status						
Never married	ref			Ref		
Currently married	0.53	0.346–0.817	0.004	0.46	0.305–0.722	0.001
Post marriage	1.07	0.663–1.757	0.758	0.89	0.546–1.442	0.632
Education level						
None	ref			Ref		
Primary (1–4)	0.78	0.514–1.188	0.249	0.97	0.604–1.579	0.924
Primary (5–7)	0.72	0.579–0.901	0.004	0.89	0.703–1.137	0.363
Secondary and tertiary	0.44	0.258–0.738	0.002	0.62	0.388–1.001	0.051
Earning money						
No	ref			Ref		
Yes	0.8	0.614–1.056	0.118	0.66	0.494–0.887	0.006
Taking alcohol						
No	ref			Ref		
Yes	0.28	0.166–0.495	< 0.001	1.26	0.794–1.995	0.328
Number of living children	1.23	1.163–1.300	< 0.001	1.34	1.260–1.423	< 0.001

## Discussion

### Key results

Nearly one-third of women aged 15–49 years who participated in the 2012 and 2017 surveys had unmet needs for modern contraception. The unmet need for modern contraception was consistently lower in HIV-uninfected women than in WLHIV-infected women. The study has revealed women sub-populations whose need for modern contraception has not been met. These are young and older women, women who have never been to school and women who are divorced, widowed or separated.

### Interpretation

The levels of unmet need for modern contraception were higher than the designated WHO cut-off point. According to the WHO, if the percentage of unmet needs is higher than 25%, then public health interventions specific to subpopulations in need of modern contraception will be highly needed [25].

We hypothesized that increased ART use might impact the levels of unmet need for modern contraception in WLHIV. The Universal HIV test and Treat (UTT) policy was implemented in Tanzania and became operational in the study setting in 2016. The 2012 and 2017 surveys were conducted before and after the UTT policy. Our study did not show an association between HIV infection and unmet need for modern contraception in the 2017 survey. Although the levels of unmet need for modern contraception were consistently higher in WLHIV than in HIV-uninfected women in both surveys, the lack of association in the 2017 survey might suggest the influence of increased ART use. We recommend studies to investigate the impact of ART on the unmet need for modern contraception by examining individual-level ART data.

Education on sexual and reproductive health for women in their reproductive years is key to promoting access to family planning and the ability to make reproductive choices. Number of years at school might be a proxy indicator for sexual and reproductive health knowledge and education. Our study has revealed high levels of unmet need for women who have not attended school, and it tends to decrease with increasing level of education. We recommend public intervention to address equitable access to sexual and reproductive services, including family planning, to include women with limited levels of education.

When the analysis was repeated in 2017, the effect of education seems to disappear. Education was no longer associated with an unmet need for contraception. In contrast, a social economic indicator variable started to appear in the equation. In 2027, women who were considered bread winners at the family level at the time of data collection were found to have a reduced level of unmet need for modern contraception by 44%. This finding suggests an influence of women’s education on fertility and family planning measures, as suggested by the authors in their previous publication [26].

### Comparison with other studies

Our research findings are supported by other studies conducted in Sub-Saharan African countries [2, 6, 27]. However, some results are conflicting with ours [28]. The main sources of variation within SSA studies are the social context of the studied community, methods of data collection and how desire for fertility was calculated. Variations in the estimates are also caused by the characteristics and sizes of the sampled study populations [6, 27].

### Study strengths and limitations

We claim to analyse data from the huge sample size. The sample size was adequate to person different comparisons with enough power. The generalizability of the findings is limited to communities in Tanzania with the same characteristics in terms of culture as those of the Mwanza region. We recognize some study limitations: unavailability of individual-level ART data to substantiate the role of ART on the levels of unmet need for modern contraception. However, we were not able to include many variables that were indicated in other studies to be addressed in family planning and modern contraception research. Finally, we were not able to look into the data longitudinally. The two surveys were not analysed longitudinally due to luck of linkage between surveys, and the 2017 survey recruited a larger set of participants who did not participate in the previous survey (2012). Previous analysis showed the cohort population is homogenous. Village was not considerd a cluster in this analysis.

## Conclusion

The Government of Tanzania recognizes the importance of providing all women with access to modern contraception, regardless of their HIV status. To better understand the current situation and to inform policy decisions, our surveys showed that there is a significant level of unmet need for modern contraception among women in Tanzania, regardless of their HIV status. Factors such as age, parity, marital status, and HIV status were identified as being associated with unmet need. In light of these data, the Government of Tanzania should increase its commitment to ensure that all women in the country have access to modern contraception, focusing on women sub-populations whose need for modern contraception has not been met.

We recommend a family planning program to address the need for these women to advance progress towards achieving university access to reproductive health. We recommend research to explain the reasons for higher levels of unmet need for modern contraception in female sub populations and WLHIV.

## Data Availability

Data cannot be shared in public but will be available upon request and approval by the Medical Research Coordinating Committee of the National Institute for Medical Research (NIMR) in Tanzania. The ethical review board approved this research work under the MRCC guidelines. The MRCC demands that all data collected within Tanzania may not be transferred or shared without their permission and before the signing of a data transfer agreement. Researchers who meet the criteria for access to the data should use the contract details below to request the data: The Secretary, Medical Research Coordinating Committee (MRCC), National Institute for Medical Research, 2448, Barrack Obama Road, P o Box 9653, Dar es Salaam, Tanzania. E-mail; ethics@nimr.or.tz.
